# Cross Talk Between Natural Killer T and Dendritic Cells and Its Impact on T Cell Responses in Infections

**DOI:** 10.3389/fimmu.2022.837767

**Published:** 2022-02-03

**Authors:** Lei Zhao, Xi Yang

**Affiliations:** ^1^ Departments of Immunology and Medical Microbiology and Infectious Diseases, Max Rady College of Medicine, University of Manitoba, Winnipeg, MB, Canada; ^2^ Laboratory of Basic Medical Science, Qilu Hospital of Shandong University, Jinan, China

**Keywords:** dendritic cell, NKT cell, infection, cross-talk, innate immunity, T cell immunity, *Chlamydiae*, NK cell

## Abstract

Both innate and adaptive immunity is vital for host defense against infections. Dendritic cells (DCs) are critical for initiating and modulating adaptive immunity, especially for T-cell responses. Natural killer T (NKT) cells are a small population of innate-like T cells distributed in multiple organs. Many studies have suggested that the cross-talk between these two immune cells is critical for immunobiology and host defense mechanisms. Not only can DCs influence the activation/function of NKT cells, but NKT cells can feedback on DCs also, thus modulating the phenotype and function of DCs and DC subsets. This functional feedback of NKT cells on DCs, especially the preferential promoting effect on CD8α+ and CD103+ DC subsets in lymphoid and non-lymphoid tissues, significantly impacts the systemic and local adaptive CD4 and CD8 T cell responses in infections. This review focuses on the two-way interaction between NKT cells and DCs, emphasizing the importance of NKT cell feedback on DCs in bridging innate and adaptive immune responses for host defense purposes.

## Introduction

The immune system comprises two arms of practical responses, innate and adaptive immunity. The innate immune system is the first line of host defense characterized by rapid responses of NK cells, NKT cells, macrophage, and DCs, while the adaptive immune system involves the clonal expansion of antigen-specific T and B cells. Most infections can induce both innate and adaptive immune responses. For adaptive T cell responses, both CD4 and CD8 T cells participate in protection from infections and disease. The importance of type 1 and type 2 T cell balance has long been recognized for determining protection or pathology in infectious diseases. DCs are the most efficient antigen-presenting cells (APCs) in priming and directing T cells, thus providing a critical linkage between innate and adaptive immunity. Different DCs modulate the differentiation/function of T cells differently, depending on their subset, phenotype, maturation, and cytokine patterns. The advances in modern immunobiology include identifying numerous non-classical, innate-like immune cells, one of which is the NKT cell. NKT cell is named initially based on their co-expression of receptors from two different types of immune cells, i.e., T cell receptor (TCR) of T cell and NK cell. NKT cells originate from the thymus and distribute to different tissues after initial development. The distribution of NKT cells in various tissues is often related to their expression of surface markers, primarily chemokine receptors and integrins involving its homing to and residing at the particular tissues. The organs with a relatively heavy residence of NKT cells include the liver, lung, adipose tissues, and intestine. A striking feature of NKT cells is that these cells can rapidly secrete large amounts of cytokines upon activation, thus quickly modulating immune responses and directly participating in host defenses in the early stage of infections and harm exposure. Increasing studies suggest a close cross-talk of NKT with DCs in the lymphoid and non-lymphoid tissues, and this cross-talk has a significant impact on CD4 and CD8 T cell responses to infections and the outcome of infectious diseases (1, 2). This review summarizes and analyzes the recently reported findings on the reciprocal interaction between NKT and DC cells and the consequent T cell responses.

## Characteristics of NKT Cells and Their Functional Subsets

After nearly three decades of study, significant progress has been made in clarifying this unique T cell population in terms of originality, differentiation, migration, activation, and functional subsets (3–6). Two major types of NKT cells have been identified based on the diversity of their TCRs. Type 1 NKT, also called classical NKT or iNKT, expresses a semi-invariant αβ TCR while type 2 NKT cells have diversified TCRs, also called dNKT (7). The semi-invariant αβ TCR used by type 1 NKT (iNKT) cells express α chain with Vα14 (in mice) or Vα24 (in humans) and Jα18. Unlike conventional T cells, NKT cells recognize glycol- and phospholipids in the context of CD1, a non-classical MHC class I molecule. Although human CD1 molecule has a, b, c and d isoforms, in both humans and mice, only CD1d is the isoform recognized by NKT cells. The lipid antigens recognized by NKT cells include endogenous and exogenous sources. Virtually all mouse (Vα14) and human (Vα24) type 1 NKT cells can recognize α-galactosylceramide (α-GalCer), a molecule extracted initially from marine sponges (8, 9) in the context of CD1d. In contrast, the TCRs of type 2 NKT cells are not responsive to α-GalCer (10). The so far identified lipid antigens include sphingolipid, glycerolipids, and phospholipids (11, 12). It is not surprising that the different NKT cells recognize various lipids considering their difference in TCR diversity. Studies found that the innate (toll-like receptor, TLR) and adaptive (TCR) signaling pathways may have influences on the function of dNKT cells (7). In terms of function, many recent studies have suggested a trend of the higher likelihood of type 2 NKT cells playing an immune-regulatory role than type 1 NKT cells *in vivo*. The type 1 and type 2 NKT cells can also cross-regulate their function (5). The feature of NKT cells for a quick response with a large amount of cytokine production (13, 14) to stimulations and the rich residence in local tissues equips them the unique capacity to bridge innate and adaptive immunity in the peripheral locations of infection. The study on type 1 NKT cells is much more extensive than type 2 NKT cells, largely due to the availability of two advanced tools for experimental investigation, i.e., the Jα18 gene knockout (KO) mice and lipid-loaded CD1d tetramers. These tools allow the specific identification and functional characterization of iNKT cells in *in vivo* settings. Thus, the type 1 NKT is the primary focus of this review.

The alterations of NKT cells, more often showing activation and expansion but sometimes exhibiting reducing number and function, in the thymus and peripheral tissues have been observed in settings including infections, cancers, autoimmunity, and allergic diseases. Recent studies have clearly shown the significant involvement of NKT cells in the protective immune responses and sometimes pathological processes. The potential to use the non-polymorphic nature of CD1d, which presents lipid antigen to NKT cell, thus avoiding graft-versus-host diseases after adoptive cell transfer, has significant implications in cellular cancer immunotherapy (7, 15). The importance of NKT cells in maintaining tissue homeostasis has also started to be recognized (16).

Studies using α-GalCer have shown a very dynamic NKT response following its activation. The process involves a significant turnover of TCR, surface marker down-regulation, and even a certain degree of apoptosis, especially in the first 24 hrs (17, 18). Since most of the studies carried out in earlier years had used anti-TCR and anti-NK1.1 antibodies to identify NKT cells, it was thought that NKT cells decrease *in vivo* in the early stages of activation following stimulation because the NK1.1+ T cell population appeared to be reduced within the first 24-48 hrs. This misperception was corrected later when CD1d tetramer became available. It was later found that although the expression of NK1.1 marker indeed reduced at the beginning of activation, NKT cells actually kept expanding in the period of 6-72 hrs after stimulation when it was examined by lipid-loaded CD1d tetramer (17, 18). The chemical modification of α-GalCer can alter NKT cell responses, particularly in cytokine production (19, 20), suggesting a potential way to manipulate NKT cell function for disease prevention or therapeutic purposes.

Subsets of NKT cells with different cytokine profiles and functions have been found in various organ tissues (4, 21–23). As summarized in **Table 1**, NKT cell subsets can be grouped into four major categories mainly based on cytokine patterns, in many ways like classical CD4 T cell subsets. NKT1, like Th1 cell, mainly produces IFN-γ and expresses T-bet; NKT2, like Th2, produces IL-4, IL-5, and IL-13 and expresses high PLZF; NKT17, like Th17, produces IL-17 and IL-22, and expresses RORγt. Moreover, IL-10-producing NKT (NKT10) cell, similar to the regulatory T cell, has also been identified (24). Notably, unlike Th1 cells, NKT1 also produces IL-4, although IFN-γ is more dominate. The dominance of specific NKT cell subsets in particular organ tissues is often seen, e.g., NKT1 often dominates the liver while NKT2 dominates adipose tissues while a mix of different subsets is more often seen in lung and intestine.

**Table 1:** Subsets of NKT cells with different cytokine profile and function.

	NKT1	NKT2	NKT17	NKT10
Cytokine profile	IFN (IL-4)	IL-4, IL-5	IL-17	IL-10
		IL-9, IL-13	IL-21	
		IL-10,	IL-22	
Transcriptional factors	T-bet	PLZF	RORγt.	
	PLZF	GATA3	PLZF	
	GATA3		GATA3	
Resident tissues	Liver, lung	Adipose tissue	Lung	
	Spleen	Lung	Skin	
		Intestine	Lymph nodes	

NKT cell subsets can be grouped into four categories mainly based on their cytokine patterns and function, similarly like classical CD4 T cell subsets. NKT1, like Th1 cell, mainly produces IFN-γ and express T-bet; NKT2, like Th2, produces IL-4, IL-9 and IL-13 and express high PLZF; NKT17, like Th17, produces IL-17 and IL-22 and express RORγt, and NKT10, like regulatory T cell produce IL-10. Unlike Th1 cell, NKT1 also produces IL-4, although IFN-γ is more dominate. The dominance of certain NKT cell subsets in particular organ tissues is often seen, e.g. NKT1 often dominates the liver while NKT2 dominates adipose tissues while a mix of different subsets is often seen in lung and intestine.

Following the initial development of effector subsets in the thymus, NKT cells, after distributing to different organs, can further experience post-thymic differentiation and preferential expansion of particular subsets according to the infection or disease settings in mice and humans. The post-thymic acquired/enhanced polarization of cytokine patterns and the development of designated NKT subsets are related to exposure to CD1d in the periphery with enhanced expression of NK cell markers such as NK1.1 (mouse) and CD161 (human). It is also suggested that although relatively mature NKT cells and NKT subsets do exist in the thymus, the NKT cells that are newly distributed to peripheral organs are more likely not fully mature, thus relying on local activation/stimulation for maturation and functional development. Once arriving at the local tissues, NKT cells usually become long-term resident, non-circulating cells. Therefore, the local microenvironment, including infectious agents and cytokines/chemokines, can significantly impact the fate of the NKT cells. A recent study using RNA-seq and ATAC-seq analyses showed that compared to iNKT cells in other organs, lung iNKT cell subsets exhibit the most distinct location-specific features, shared with other innate lymphocytes in the lung (25). The study also shows that iNKT cells undergo chromatin and transcriptional changes upon activation, leading to two subpopulations: one is similar to follicular helper T cells while the other is more NK- or effector- like. The study suggests the importance of chromatin remodeling in the formation of NKT subsets.

## The Activation of NKT Cells and the Role of DCs

The activation of NKT cells by infections is initiated with the presentation of microbial lipid antigens (sometimes endogenous lipids) by the antigen-presenting cells (APCs), which express CD1d, the MHC-I-like molecule on the cell surface. The APCs for NKT include, but are not limited to, macrophages, conventional DCs, and monocyte-derived inflammatory DCs. When APCs take up microbial organisms, microbial or endogenous lipid antigens are released and loaded onto CD1d, consequently activating NKT cells through TCRs. Compared to other APCs, DC cells express much richer surface costimulatory molecules and produce more immunomodulatory cytokines, thus most powerful in promoting NKT cell activation and post-thymic polarization. Many experimental studies have shown the interaction between DCs and NKT cells at the phase of NKT activation when model antigens and infectious agents are tested. The studies have shown a critical role of DCs in the activation of NKT cells in various scenarios. In general, three potential pathways might be involved in DC-mediated NKT cell activation and polarization following infections (26, 27):1. CD1 molecules can present microbial lipid antigens on DCs, directly activating NKT cells.2. DCs activated by the microbial ligands for pattern recognition receptors (PRRs), such as toll-like receptors (TLRs), can produce immunomodulatory cytokines and present host endogenous lipid antigens on their surface CD1 molecules, consequently activating NKT cells without the involvement of microbial lipids.3. The ligands expressed by microbes can activate DCs through PRRs, leading to the production of proinflammatory cytokines, which can directly activate NKT cells without the engagement with TCRs recognizing lipid antigens.

Numerous microbial lipid antigens have been identified for their capacity to trigger type 1 or type 2 NKT responses (6, 28). *Sphingomonas spp* are probably the first group of Gram-negative bacteria colonizing the mucosal surface of mice and humans to be found to express cell wall lipid antigens that can activate NKT cells. It was reported that these bacteria abundantly express glycosphingolipids (GSLs) which have high similarity in structures to α-GalCer. Our group studied the role of NKT cells in host defense against *Chlamydiae*, an intracellular bacterial pathogen that poses a threat to public health worldwide (29, 30). We found that chlamydial glycolipid exoantigen (GLXA), a glycolipid antigen derived from *Chlamydia muridarum*, can activate iNKT cells at *in vitro* and *in vivo* settings. We showed that GLXA specifically stimulated iNKT1.4 hybridoma cells to produce IL-2 and activated primary iNKT cells to produce various cytokines in a CD1d-dependent manner (31). Diacylglycerols (DAGs) are another type of bacterial antigens that can activate NKT cells. DAGs are found in *Borrelia burgdorferi*, *Streptococcus pneumonia*, *Corynebacterium glutamicum*, *Mycobacterium tuberculosis*, *Listeria monocytogenes*, and *Mycobacterium smegmatis* (6).

In addition to bacteria, lipid antigens from fungus and protozoan parasites were also reported to induce NKT responses (32), but no virus lipid antigen has been found, although the activation of NKT cells are well documented in viral infections (33, 34). Moreover, in the condition of bacterial infections, endogenous lipid antigens such as isoglobotrihexosylceramide (iGb3) and β-glucosylceramide could also possibly participate in the activation and regulation of NKT response (35). On the other hand, the microbial lipids antigens recognized by type 2 NKT cells, which exhibit diversified TCRs, appear different from those recognized by type 1 NKT cells (5). These type 2 NKT cells preferably recognize lipid antigens of phosphatidyglycerol (PG), diphosphatidyglycerol (DPG) and phosphatidylinositol lipids (36, 37). Although either innate signaling such as TLR activation and adaptive TCR signaling through APCs, especially DCs, can activate NKT cells, it is likely that, in most circumstances, both signaling pathways are involved.

## Modulation of DC and DC Subsets by NKT Cells

The interaction of NKT cell and DC is reciprocal. More and more studies suggest that NKT cells receive activation signals from DCs and often feedback on DCs. The earlier studies mainly used model antigens to test the influence of NKT cells on the function of DCs (38–43). Fuji et al. showed that a single i.v. injection of α-GalCer promoted the maturation of splenic DCs, leading to a marked increase in the surface expression of costimulatory and MHC Class II molecules with enhanced antigen presentation function (39). They saw that α-GalCer did not directly influence DCs; rather, it did it through activation of NKT cells. Also using α-GalCer as an NKT stimulator, Stober et al. found that NKT cells could help DCs initiate antiviral cytotoxic T cell (CTL) responses in an MHC Class I-dependent manner (38). An interesting finding in the study was that, for the DCs to obtain help from NKT cells for activation of CTL, the same DCs had to present different antigens (glycolipid and peptide), respectively, for NKT and CTL cells. Notably, the study on the adjuvant effect of NKT or NKT activating lipid antigens to promote DC function represents a significant effort to improve vaccine efficacy

Over the past decade, we did a series of studies on NKT cell responses in respiratory tract *Chlamydia muridarum* and *Chlamydia pneumoniae* infections, particularly on the feedback of NKT cells on DC function (29, 44–48). NKT cell responses in human chlamydial diseases have also been reported (49). We have demonstrated that NKT cells, particularly type 1 NKT (iNKT) cells, play a crucial role in host defense against chlamydial infections, especially in *C. pneumoniae*-caused infection. Variation in iNKT cells for cytokine production was also observed in infections of different strains of *Chlamydiae* (50). iNKT cells can promote protective type-1 immune responses to *C. pneumoniae* by inducing enhanced IFN-γ-producing Th1/Tc1 type CD4+/CD8+ T cells and IL-17-producing Th17 cells in the spleen and the local tissues in the lung (45). Our study has used various experimental approaches, including comparing wild-type (WT) and NKT cell-deficient, Jα18 gene knockout (KO) mice, the adoptive transfer of purified NKT cells to NKT deficient mice, and the enhancement of NKT activation by α-GalCer. We demonstrated that NKT cells could significantly influence splenic and pulmonary DCs in their phenotype, cytokine pattern, subsets, and function for modulating CD4 and CD8 T cell responses and the isotypes of antibody (44, 45, 47). Targeted analysis of NKT cell cytokine patterns using lipid-loaded CD1d tetramer and intracellular cytokine staining showed that the NKT cells activated by respiratory tract *C. pneumoniae* infection predominantly produced IFN-γ, which correlated well with the enhanced type-1 responses of both CD8+ and CD4+ T cells (50). *In vitro* co-culture of splenic DCs with NKT cells enhanced bioactive IL-12p70 production by DCs in a CD40L, IFN-γ, and cell-cell contact-dependent manner. Furthermore, DCs isolated from infected wild-type (WT) and iNKT deficient mice induced type-1 and type-2 T cell responses, respectively, when the DCs were co-cultured with T cells *in vitro* or adoptively transferred to naïve mice *in vivo*. Studies on lung DCs also showed significantly altered number, phenotype, and cytokine profile of lung DCs in iNKT deficient mice following *C. pneumoniae* infection. The lung DCs from infected iNKT deficient mice failed to promote type 1 T cells *in vitro* and *in vivo*, which was associated with failure in inducing protection to challenge infection (47). The finding of modulating effect of NKT cells on the function of DCs in local tissues provides more relevant insight into the interaction of NKT and DC cells in infection. The results provide direct evidence on the functional modulation of NKT cells on systemic and local DCs in infections.

The studies on DC function have led to the identification and characterization of different DC subsets, which, by nature or through post-thymic development in the microenvironments, express variable surface makers and produce different cytokines related to DC migration and cell interaction and function. Many DC subsets have been reported in humans and mice. Conventional DCs (myeloid and lymphoid), abbreviated as cDC and plasmacytoid DCs (pDCs) are the two large groups of DC subsets. CD11c is the most often used marker for mouse conventional DC cells. CD8α+ and CD8α- DC subsets are a common grouping in studying conventional splenic DCs with implications in functional differences (51). Indeed, many studies have suggested the distinction of the CD8α+ and CD8α- DC subsets in function although inconsistent data were also reported (52, 53). The difference in costimulatory surface markers and cytokine patterns is often related to the functional distinction (54–57). Notably, DC subsets in peripheral tissues with different surface markers have been better characterized due to improved technology, especially the advances in multi-color flow cytometry of small cell populations (58–60). Based on the differential expression of CD11b and CD103 molecules, mouse pulmonary DC can be sub-grouped into CD11bhighCD103- and CD11b-/lowCD103+ DC subsets (59, 60). Interestingly, CD103+ DCs in the non-lymphoid organs, such as the lung, gut, and skin, form a unified subset that is developmentally related to the CD8+ cDC in lymphoid organs (61). This correlation is demonstrated by their shared dependence on certain transcriptional factors such as Batf3 and Irf8 and functional characteristics of antigen cross-presentation. This close linkage of CD8+DC and CD103+DC was further strengthened by the reports showing these DC subsets’ unique common expression of XCR1, a chemokine receptor (62, 63). Since both human BDCA3+ DCs and sheep CD26+ DCs, which are the equivalents of mouse CD8+ DCs, also express XCR1, it is suggested by the researchers to name “XCR1+DCs” to designate the “CD8+ type DCs” in both lymphoid and peripheral tissues across all mammalian species and tissues. Functionally, the similar difference of CD103+ and CD103- pulmonary DC subsets and CD8α+ and CD8α- splenic DC subsets, in cross-presentation of exogenous antigens has been found (60). Our study on splenic DCs has demonstrated significantly stronger capacity for splenic CD8α+ DCs and pulmonary CD103+DCs in inducing protective immunity against chlamydial lung infections (64, 65). When the effect of NKT cell on splenic and pulmonary DCs was examined, we found a strong promoting effect of NKT cell on the DC1-like DCs and a preferential modulating effect of NKT cells on CD8α+ DCs in number and function (44, 47). We showed that CD8α+ DCs in the NKT deficient mice expressed lower CD40 and produced less IL-12. Functionally, co-cultured naïve CD8α+DCs with NKT cells from chlamydial infected mice promoted IL-12p70 production by this DC subset in a CD40:CD40L interaction-dependent manner. Consistently, CD8α+ DC from Jα18 KO mice showed significantly reduced ability to induce type 1 T cell immunity and protection *in vivo*, compared with those from WT mice. The modulating effect of NKT cells on DCs was also confirmed by the adoptive transfer of NKT cells to Jα18 KO NKT deficient mice. A similar preferential modulating effect of NKT cells on CD8α+ DC subset was also found in leishmanial infection (44).

Besides its direct effect on DCs, NKT cells can also influence DC function through other types of innate immune cells, such as NK cells. We reported a decade ago that NKT cells, following chlamydial infection, can influence the function of NK cells and NK subsets (46). In addition, we found then that NK cells could influence DC function for the induction of Th1 cells in chlamydial infection (66). Our recent work showed that NK cells could significantly modulate DC function in the induction of type 1 T cell development and the inhibition of regulatory T cells (67). We showed that pre-depleting NK cells significantly impaired type 1 T cell responses to the infection, but contrarily enhanced FOXP3+ Treg cells and IL-10-producing CD4 T cells, leading to enhanced disease severity and chlamydial growth in the lung (68). NK cell-depleted mice showed decreased Th1 and Th17 cells, which was correlated with reduced IFN-γ, IL-12, IL-17, and IL-22 production, as well as T-bet and RORγt expression. NK cells can modulate the surface molecule expression and cytokine production profile. The adoptive transfer of DCs from NK cell-depleted mice showed reduced induction of type 1 CD4 and CD8 T cells but enhanced FOXP3+ Treg cells and IL-10-producing CD4 T cells. Consistently, the recipients of DCs from NK cell-depleted mice failed to be protected against chlamydial lung infection. Mechanistically, we found that NK activating receptors, surface costimulatory molecules, and cytokines produced by NK cells play a significant role in the modulating effect of NK on DC function (69). In the NK cell and lung DC co-culture experiments, we found the blockade of the NKG2D receptor reduced the production of IL-12p70, IL-6, and IL-23 by DCs. The neutralization of IFN-γ decreased the production of IL-12p70 by lung DCs, whereas the blockade of TNF-α resulted in diminished IL-6 production. Considering the much large population of NK cells in local tissues, including the lung, the NK promoting/modulating effect of NKT cells could indirectly amplify their impact on DC function. Therefore, the coordination of NKT and NK cells may have a broader impact on other immune cells, which involve the link between innate and adaptive immunity in infections.

To examine the interaction of NKT, NK, and DC cells more directly, we recently investigated the influence and mechanism of iNKT cells on the differentiation and function of NK cells in chlamydial lung infection and the role of DCs in this process (46). We found that the quick expansion of IFN-γ-producing NK cells following chlamydial infections did not happen in iNKT deficient mice. The expression of activation markers and the production of IFN-γ by different NK subsets were significantly lower in the iNKT deficient mice. We further found that the activation of NK cells was delayed when they were co-cultured with DCs from iNKT deficient mice, and the adoptive transfer of DCs from the deficient mice induced lower NK cell activation and less IFN-γ production by T cells. The results provide evidence on the critical role played by DCs in the modulating effect of iNKT cells on NK cell function. Not only influencing primary T cell responses but NK cells were also found to influence memory T cell responses (70). Based on the reports showing the modulating effect of NK on DCs (66, 67, 69), the findings also suggest a reciprocal influence between DC and NK cells. In addition, considering the massive evidence of NK-mediated promotion/activation of DCs, the enhanced NK cell function can further promote DC function, forming a positive feedback circle. Notably, the influence of NK cells could reach multiple cell types. For example, we recently found NK cells can influence macrophage polarization in the lung following chlamydial infection (71). Similarly, the newly reported molecules such as SND1 and Sema3E, which modulate DC functions in infections (72, 73), would be an area for exploring the molecular mechanisms related to the modulating effect of NKT and NK cells on DC function infections. The data suggest the complexity of multiple innate and adaptive immune cell interaction levels in infections.

On the other hand, a negative modulatory role of NKT cells on DC function was also reported in multiple physiological and pathological settings, especially for dNKT cells (5, 74, 75). In some circumstances of tumors, autoimmune diseases, and infections, the role of iNKT and dNKT appears opposite to the disease process in which dNKT cells are more likely showing an effect of immune suppression, although many exceptions have also been reported (5). In autoimmune diseases, dNKT cells more likely play a protective role due to the tolerogenic nature of the cell. For example, sulfatide reactive dNKT cells in experimental autoimmune encephalomyelitis (EAE) can induce tolerized DCs leading to anergy of type 1 NKT cells and inhibition of pathogenic autoreactive CD4 T cells (76, 77). However, the regulatory role of iNKT cells on DCs is also documented, and the microenvironments, especially cytokines, have a significant impact on the iNKT function. For example, Bochtler et al. reported that the competence of DCs to prime proinflammatory CD8 T cell responses was impaired by iNKT cells only in the presence of type 1 IFN (74). In addition, Caielli et al. found that the same iNKT cell could be a positive or negative regulator of myeloid DCs depending on the presence or absence of specific molecules which simultaneously acted on the DCs (75). In the absence of TLR4 co-stimulation, NKT cells triggered immature DC to become tolerogenic DC, consequently inducing regulatory T cells to prevent autoimmune diabetes, while in the presence of TLR4 co-stimulation, this tolerogenic effect was not observed. Not surprisingly, the positive and negative modulating effects of NKT cells were mediated through distinct signaling pathways. For the induction of tolerogenic DCs, the ERK1/2 pathway was taken, while for proinflammatory DCs, the NF-κB pathway was activated (75). Therefore, although dNKT appears to have a higher chance to play an immune-regulatory role, the function of different NKT cell types in disease settings is more likely determined by multiple factors including, but not limited to, their current molecular expression, maturation stage, and cytokine microenvironments.

## Concluding Remarks

**Figure 1:**
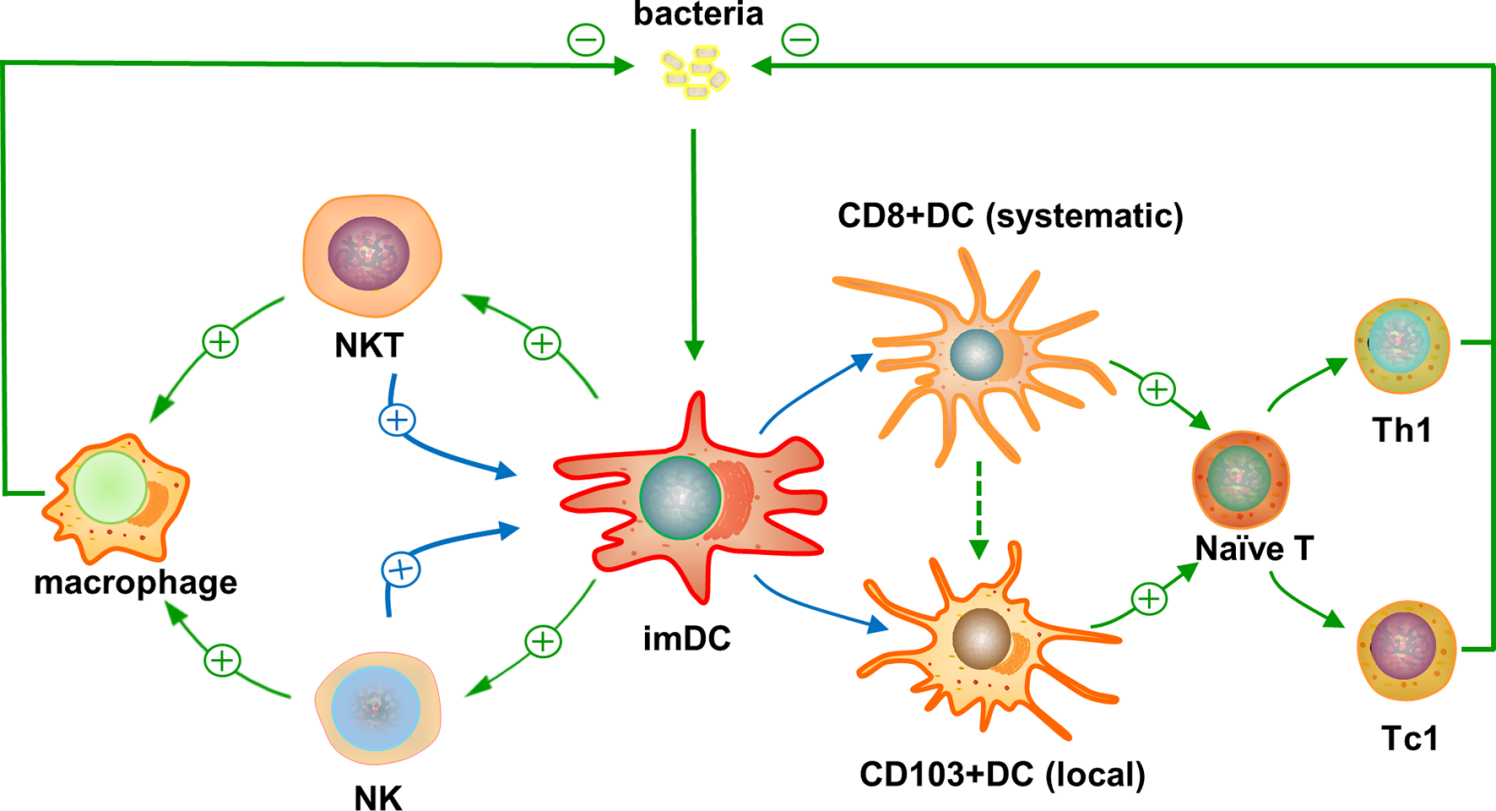
Reciprocal interaction of natural kill T (NKT) cells and dendritic cells (DCs) and its impact on T cell responses against infections. The entered bacterial or some other infectious agents are taken by immature DCs and their microbial lipid antigens are presented through CD1d to activate NKT cells. The activated NKT cells can feedback on the DCs promoting their maturation and preferential differentiation to CD8α+ DCs, systemically, and CD103+ DCs in local tissues. Possible molecular and functional link between CD8α+ and CD103+ DC subsets has been suggested. The preferentially-promoted DC subsets can direct the differentiation of conventional CD4+ or CD8+ naïve T cells into functional Th1- or Tc1-like peptide antigen-specific T cells, respectively, due to their predominant expression of co-stimulatory surface markers and production of cytokines for type 1 T cell responses. The activated antigen-specific CD4+ Th1 and CD8+ Tc1 T cells can inhibit the infectious agents locally and systemically. In addition, the feedback of NKT cells on immature DCs can lead to enhanced activation of NK cells, which also have positive feedback effect on DCs for their function to promote type 1 T cell responses. This indirect interaction of NKT and NK cells through DCs can amply the positive feedback of NKT cells on DC function. Moreover, the activated NKT and NK cells can promote the function of local monocytes and macrophages to inhibit the infection. Symbols and abbreviations: ⊕, promote; ⊝, inhibit; imDC, immature DC; naïve T, naïve T cell; Th1, CD4+ type 1 T cell; Tc1, CD8+ type 1 T cell.

Since its relatively-new identification about two decades ago, NKT cell is gradually recognized as a small but critical innate-like T cell component in the central and peripheral immune systems, playing an essential role in host defense and immune regulation. Recent studies show that NKT cells can significantly influence the phenotype, subsets, and function of systemic and local DCs (**Figure 1**). Through its modulating effect on DCs, NKT cells play a critical role in bridging innate and adaptive immunity, especially for T responses. Reported studies, especially those in real disease animal models and humans, have documented either positive or negative modulating effects of NKT cells on DC function, but more studies showed positive effects, especially in infectious diseases. NKT cells not only receive DC’s help for their initial activation but also feedback on DCs later for their functional maturation and subset development/expansion, especially for systemic CD8α+ and peripheral CD103+ DCs. The DCs modulated by NKT cells can further influence the activation/differentiation of conventional CD4+ and CD8+ T cells, which are critical for host defense mechanisms. In addition, NKT cells can promote the function of NK cells, which directly play a protective role in infections and boost the function of DCs, leading to amplified positive feedback effects on DCs. Considering the extreme importance of DCs in directing T cell immune responses, it would be necessary in future studies to focus on the interaction between NKT cells and DCs in the phases of both NKT-DC and DC-T interactions, especially the involved surface and intracellular molecules and signaling pathways by which NKT cells modulate DC/DC subset function. This knowledge is beneficial not only for a better understanding of immune regulation mechanisms, including the immunobiology of NKT and DC cells but also beneficial for rational development of vaccines through selective targeting and manipulating NKT and DC subsets to promote the protection and reduce potential immunopathology, particularly in infectious diseases and broadly in other diseases including autoimmunity and cancer.

## Author Contributions

XY and LZ contributed to article writing, figure drawing, and formatting. All authors contributed to the article and approved the submitted version.

## Funding

This work was supported by grants from the Canadian Institutes of Health Research (CIHR) to XY (CCI 92213, MOP-130423), National Natural Science Foundation of China to LZ (nos. 81501761) and Project ZR2020MH303 supported by Natural Science Foundation of Shandong Province to LZ. XY was the Canada Research Chair in Infection and Immunity.

## Conflict of Interest

The authors declare that the research was conducted in the absence of any commercial or financial relationships that could be construed as a potential conflict of interest.

## Publisher’s Note

All claims expressed in this article are solely those of the authors and do not necessarily represent those of their affiliated organizations, or those of the publisher, the editors and the reviewers. Any product that may be evaluated in this article, or claim that may be made by its manufacturer, is not guaranteed or endorsed by the publisher.
